# PDBFS: A novel active routing protocol for prioritized delivery of safety messages in VANETs

**DOI:** 10.1371/journal.pone.0354247

**Published:** 2026-08-21

**Authors:** Waqar Shah, Shahab Haider, Noreen Khan, Fasee Ullah, Arfat Ahmad Khan, Mohammed M. Alammar, Z. M. S. Elbarbary

**Affiliations:** 1 Department of Computer Science, City University of Science and Information Technology, Peshawar, Pakistan; 2 TeleCoN Research Center, Faculty of Computer Science and Engineering, Ghulam Ishaq Khan Institute of Engineering Sciences and Technology, Topi, Swabi, Pakistan; 3 Department of Computing, Universiti Teknologi PETRONAS, Seri Iskandar, Malaysia; 4 Department of Computer Science, College of Computing, Khon Kaen University, Khon Kaen, Thailand; 5 Department of Electrical Engineering, King Khalid University, Abha, Asir, Saudi Arabia; 6 Center for Engineering and Technology Innovations, King Khalid University, Abha, Saudi Arabia; 7 Electrical Department, College of Engineering, Kafrelsheikh University, Kafrelsheikh, Egypt; University of Cincinnati, UNITED STATES OF AMERICA

## Abstract

Recent technological advancements have given rise to the intelligent transportation systems. This enhances the capability of nodes, through injection of intelligence, for the minimization of road accidents. To route safety messages, direction-aware greedy protocols take into account various parameters, such as current location of a node, node’s direction, and the relative positions of source and destination nodes. However, these protocols are incapable of catering for the frequent topological changes in VANETs, yielding recurring network partitions. Moreover, these protocols lack efficient mechanism for traffic load management on a route. Furthermore, the existing protocols do not provide prioritization of safety messages over non-safety messages that adversely impact their performance. To resolve these issues, this paper pioneers the use of two parameters, namely, relative speed among nodes and packet rate, in addition to the existing direction-aware greedy approach for the selection of the best possible route. Relative speed among nodes caters for the frequent topological changes on the network by selecting a route with increased lifetime, whereas packet rate enables efficient traffic load management. Moreover, we propose a novel probabilistic approach for the selection of the best route on the basis of the aforementioned parameters. This work also introduces a new method to prioritize the time-critical safety messages over non-safety messages. Simulation results demonstrate that PDBFS reduces the average packet loss rate by 6.2%, 18.1%, and 23.4% and enhances the average network throughput by 9.3%, 15.3%, and 22.0% in comparison with TDMP, AODV-R, and TDSRP-DC, respectively. Moreover, our proposed PDBFS minimizes average end-to-end delay by 2388 ms, 2914 ms, and 3362 ms and improves the average link lifetime by 3600 ms, 4101 ms, and 4307 ms in comparison with TDMP, AODV-R, and TDSRP-DC, respectively.

## 1 Introduction

Modernday technological developments have enhanced the legacy transportation system to a smarter level, which is called as Intelligent Transportation System (ITS) [1]. To this end, Vehicular Ad hoc Networks (VANETs) have emerged as one of the key enablers of ITS [2]. VANETs provide various applications, such as travel route identification, time prediction for a journey, road accidents prevention, infotainment, and building smart cities [3]. Road accidents retains the highest importance among these applications being the major contributor of deaths and injuries in human beings. Due to these accidents, each year 1.35 million deaths and 20−50 million injuries are recorded across the globe [4]. This demands significant preventive measures against road accidents.

In this regard, Cooperative Collision Avoidance (CCA) schemes have gained tremendous popularity [5]. A CCA schme calculates the probability of collision among vehicles (herein, referred to as nodes) at a given interval. Safe speed is computed for a target node when it exceeds a predefined threshold. Two types of messages are common in VANETs, i.e., safety and non-safety messages [6]. Non-safety messages are employed for infotainment, whereas a safety message intimates nodes regarding a possible accident (herein, referred to as collision). A safety message comprises collision probability and safe speed, which are employed to prevent a possible collision [2]. These messages are disseminated by employing either a Vehicle-to-Vehicle (V2V) or a Vehicle-to-Infrastructure (V2I) model. A V2V model enables direct communication among mobile nodes, whereas V2I needs deployment of roadside infrastructure and exhibits increased capital and operational cost [7,8]. It is worth-mentioning that in addition to efficient collision probability and accuracy in computation of safe speed, in-time reliable delivery of a safety message remains equally important [9]. This is because, any delays and packet losses during transmission may cause large scale collisions among nodes. Thus, in-time reliable delivery of messages also remains challenging in VANETs due to frequent topological changes.

Multiple routes to a destination are extremely certain due to immense increase in the number of nodes on networks. In such a case, when there are more than one routes from the source to destination, identification of the best route becomes a critical decision [2]. A best route exhibits minimal packet losses and end-to-end delay. In this selection process, next hop selection (herein, referred to as the best forwarder) plays a pivotal role. To this end, greedy routing protocols employ position of a relay node and selects the best forwarder on the basis of minimum distance with the destination node [10,11]. This remains effective for a network having static nodes, however, such greedy protocols lack efficiency in networks with mobile nodes. In this regard, direction-aware greedy protocols enable selection of the best forwarder that is moving in the direction of the destination node [9]. Similarly, Reliable Target Driven and Mobility Prediction (TDMP) proposed in [12], considers received signal strength index to predict mobility patterns of a forwarder and a destination node. The selection of a node, as forwarder, basis upon two parameters, i.e., distance and direction. Moreover, Ali et al. [13] propose a dependable routing protocol called Ad hoc On-Demand Distance Vector[CTRL U+0096]Reliable (AODV-R), which extends the conventional AODV protocol by incorporating reliability considerations. Their work introduces a reliability-aware model for VANETs with the aim of achieving more stable and dependable route discovery. The central concept of AODV-R is link reliability, which reflects the probability that a communication link between two nodes will remain available for a certain duration for improving route stability. Similarly, Dhanasekaran et al. [14] present the Traffic Density Stable Routing Protocol based on Connection and Distance (TDSRP-DC). This protocol is designed to minimize packet collisions at road intersections and to adapt routing decisions dynamically according to traffic conditions. TDSRP-DC relies on Vehicle-to-Vehicle (V2V) communication, allowing nodes to identify the most appropriate next junction and forward packets accordingly to form an efficient multi-hop route. To capture real-time traffic dynamics, nodes periodically exchange information with their neighbors. The routing framework consists of network formation, neighbor discovery, fitness value computation, and routing decision-making. Several parameters are considered for the selection of an optimal route, including inter-nodes distance, vehicle speed, azimuth angle, link stability, and link reliability. Despite their effectiveness in improving route stability and reliability, both AODV-R and TDSRP-DC lack explicit mechanisms for prioritizing time-critical safety messages over non-safety traffic. Direction-aware greedy protocols still have several limitations, such as these protocols (i) are incapable to cater for the frequent topological changes on the network that may cause network partitions, hence, all the packets forwarded on the selected path are dropped, (ii) do not provide an efficient mechanism to assess traffic load on a selected path, and (iii) lack the ability to prioritize safety messages over non-safety messages. These limitations adversely impact the network performance, thereby, yielding higher packet loss rate and increased end-to-end delay, and reduced throughput.

### 1.1 Novelty and contributions

To address these issues, we propose a novel Probabilistic Direction-aware Best Forwarder Selection (PDBFS) protocol having the below mentioned contributions.

1. We propose a novel probabilistic approach for the selection of the best forwarder and route on the basis of two newly added parameters, i.e., relative speed among nodes and packet rate.
2. To the best of our knowledge, PDBFS pioneers the use of the aforementioned two parameters in addition to the existing direction-aware greedy parameters. Relative speed among nodes helps to cater for the frequent topological changes on the network by selecting a route with higher lifetime, whereas packet rate enables efficient load management on the selected route.
3. This work introduces a new method for the prioritization of time-critical safety messages over non-safety messages.
4. As demonstrated in the simulation results, PDBFS reduces the average packet loss rate by 6.2%, 18.1%, and 23.4% and enhances the average network throughput by 9.3%, 15.3%, and 22.0% in comparison with TDMP, AODV-R, and TDSRP-DC, respectively. Moreover, our proposed PDBFS minimizes average end-to-end delay by 2388 ms, 2914 ms, and 3362 ms and improves the average link lifetime by 3600 ms, 4101 ms, and 4307 ms in comparison with TDMP, AODV-R, and TDSRP-DC, respectively.

### 1.2 Paper organization

Hereinafter, the paper is organized as follows. Section 2 critically reviews the related literature. The proposed protocol is detailed in Section 3. Section 4 evaluates the performance of PDBFS in comparison with eminent protocols. Finally, Section 5 provides the concluding remarks with future directions.

## 2 Related work

Frequent topological changes occur due to bi-directional high speed movement of nodes in VANETs, which degrade link quality among nodes as well as its lifetime. This results in frequent link breakages, higher end-to-end delays, and reduced packet delivery rates. This section critically reviews eminent protocols from the literature for the exchange of safety messages among nodes.

The authors in [15] propose a direction-based protocol, which takes into account link quality to find the best route. Similarly, The authors in [16] propose a protocol that estimates link reliability to enable reliable routing. Furthermore, the work in [17] proposes Prediction-based Greedy Perimeter Stateless Routing (PGPSR) protocol that uses a probabilistic approach, which estimates the route sustainability for the selection of the best possible route. Moreover, the authors in [18] present an Improved Geographic Routing (IGR) protocol that employs link error rate and nodes’ density on the particular route for the selection of the most appropriate route. In [19] the authors propose an opportunistic routing protocol, which uses a weighted algorithm to select intermediary relay nodes. This improves packet delivery rate and yields improved efficiency. However, computational overhead remains significantly high that remains one of its disadvantages. In [9] the authors propose a Direction Aware Best Forwarder Selection (DABFS) protocol, which uses direction and relative position of nodes along with distance as parameters to ensure in-time and reliable delivery of messages. However, DABFS fails to cater for efficient load management on a selected route.

In [20], the authors propose a novel protocol, namely, Compacted Area with Effective Links (CAEL). This protocol reduces communication overhead to enable reliability by removing the extraneous nodes and selection of trusted nodes. The work in [21] proposes another protocol that enables route information dissemination through *CH* that reduces network congestion. The work in [22] presents a new protocol, called Enhanced Hybrid Ant Colony Optimization Routing Protocol (EHACORP) that uses ant colony optimization to identify the shortest path for the transmission of messages. Another protocol, called Alternate Link-based Multi-path Reactive Routing (ALMRR), is proposed in [23]. ALMRR maintains multiple paths from a source to a destination, where an optimum path is identified among the available set of paths. The rest of the paths are kept as alternatives for a situation when the selected path goes down. The authors in [24] propose Weight-aware greedy protocol, named as Weight-aware Greedy Perimeter Stateless Routing (WA-GPSR). The proposed protocol computes weight for each possible forwarder on the basis of link lifetime, cumulative communication time, traffic node density, and node mobility. A node bearing the highest weight is selected as forwarder that ensures significantly efficient routing. In [25] the author integrate the features of Genetic Algorithm (GA) and Firefly Algorithms (FA) into a new algorithm, namely, Hybrid Genetic Firefly Algorithm (HGFA) for fast and reliable communication among nodes.

The work in [7] introduces another protocol, called Energy Efficient-Fast Message Distribution Routing Protocol that takes into account different features, such as node’s position, direction, and message delivery time for the selection of a forwarder. The work in [26] proposes a Dynamic trilateral Enrolment (DyTE) protocol, which selects a trilateral zone to identify the destination node. Results show that DyTE achieves higher packet delivery ratio and enhances network throughput. Moreover, the authors in [27,28] present fuzzy logic-based variants of GPSR, which integrates neighboring information of a node with link quality to find the best possible route for the transmission of messages. Furthermore, Reliable Group of nodes identifies reliable nodes for forwarding in terms of availability and geographical position to reduce communication overhead [29]. The work in [30] combines *K*-means clustering with Scatter Search (SS). In this hybrid approach, *K*-means performs clustering, whereas SS identifies the best possible route, to a destination node, among the available set of routes. Another protocol, namely, Reliable Target Driven and Mobility Prediction (TDMP), is proposed in [12]. TDMP takes received signal strength index into account to predict mobility patterns of a forwarder and a destination node. The selection of a node, as forwarder, basis upon two parameters, i.e., distance and direction. The authors in [13] proposes a dependable routing protocol, namely, Ad hoc On-Demand Distance Vector-Reliable (AODV-R), which is an enhanced version of the widely adopted AODV routing protocol. In this work, the authors introduce a novel reliability-aware model for VANETs that focuses on enabling dependable and stable route discovery. The core idea of the proposed approach is link reliability, which increases the likelihood for a communication link between nodes to remain continuously available over a given period of time. Similarly, the work in [14] introduces a Traffic Density Stable Routing Protocol based on Connection and Distance (TDSRP-DC), which is designed to reduce data packet collisions at road intersections and to dynamically adapt routing decisions. The proposed approach relies on Vehicle-to-Vehicle (V2V) communication, where nodes identify the most suitable next junction and forward data packets towards the destination to establish an optimal multi-hop route. The protocol estimates real-time traffic variations by periodically exchanging information among nodes. The overall framework involves network formation, neighbor discovery, fitness value estimation, and routing decision-making. Key parameters considered for determining the optimal route include node distance, vehicle speed, azimuth angle, link stability, and link reliability. However, both AODV-R and TDSRP-DC do not provide any mechanism to cater for the prioritization of the time-sensitive safety messages over non-safety messages.

From the review of the existing literature, it is found there are a number of protocols that intend to ensure in-time reliable delivery of messages. However, these protocols still exhibit several limitations, such as these protocols are found incapable to cater for the frequent topological changes on the network. Moreover, effective means to manage load on a selected path are also found lacking in these protocols that results in putting extra burden on a selected path. This limits the network efficiency by increasing packet losses and end-to-end delay, and reducing network throughput. To resolve the aforementioned issues, we propose a novel protocol that is detailed in the following section.

## 3 The proposed PDBFS protocol

**Fig 1:**
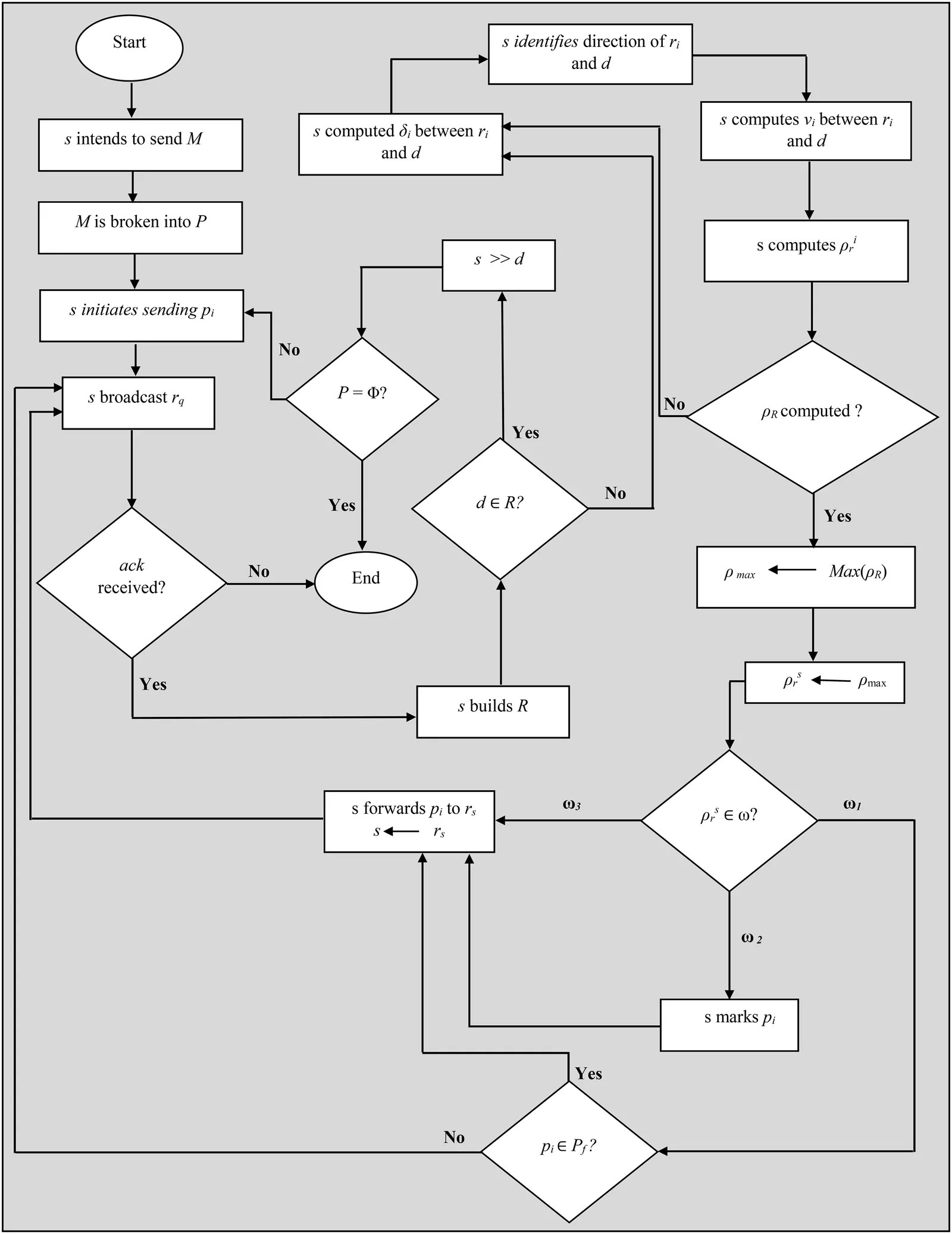
Procedural flowchart of the proposed PDBFS protocol.

This section presents the detailed design and operational methodology of the proposed Probabilistic Direction-aware Best Forwarder Selection (PDBFS) protocol. The proposed protocol introduces a novel probabilistic forwarding mechanism that intelligently selects the most suitable forwarder node based upon the relative speed among nodes and packet rate. This ensures reliable, efficient, and timely dissemination of safety messages in VANETs. By incorporating probabilistic decision-making, the proposed protocol aims to reduce packet loss, minimize transmission delay, and improve throughput and link lifetime to achieve reliability in highly dynamic networks. Fig 1 illustrates the detailed procedural flowchart of the proposed PDBFS protocol consisting sequence of operations and forwarding decisions involved during message dissemination.

Routing protocols retain a vital role in VANETs, as reliable and in-time delivery of messages is crucial, especially in time-sensitive applications. Therefore, selection of the best route acquire a significant stature in ensuring successful delivery of messages, which bears minimum end-to-end delay, fewer packet losses, and maximum possible throughput,. To this end, different researchers have proposed various greedy and direction-based protocols [9]. However, paucity to counter frequent topological changes and lacking the ability to efficiently manage traffic load on a selected path limit their efficiency. This yields increased packet losses, end-to-end delay, and reduced network throughput. To address these issues, we propose a novel protocol, namely, PDBFS, that does not only rely upon the greedy direction-aware approach. PDBFS comprises a new probabilistic approach to find the best forwarder that remains critical in identification of the most appropriate route to the destination. This approach pioneers the use of relative speed among nodes and packet rate for the selection of route. Here, relative speed among nodes helps to manage frequent topological changes, whereas packet rate prevents the route from getting overloaded. This yields minimized packet loss rate, reduced end-to-end delay, improved network throughput, and increased link lifetime of the selected route. Moreover, we introduce a new technique to prioritize safety messages over non-safety messages. The proposed PDBFS protocol comprises three algorithms, i.e., message dissemination, neighbour discovery, and the best forwarder selection, that are detailed in the following subsections.

**Algorithm 1 Message Dissemination**

**Input:** *s* and *d*

**Output:** Success or Failure of safety messages

**Begin**

Build *R* for *s*

**Repeat**

**If** *d* ∈ *R* **Then**

*s*>> *d*

**End**

**Else**

Identify $r_{s}$

*s*>> $r_{s}$

*s* ⟵ $r_{s}$

**End If**

**Until** All the packets are delivered.

**End**

### 3.1 Message dissemination

**Fig 2:**
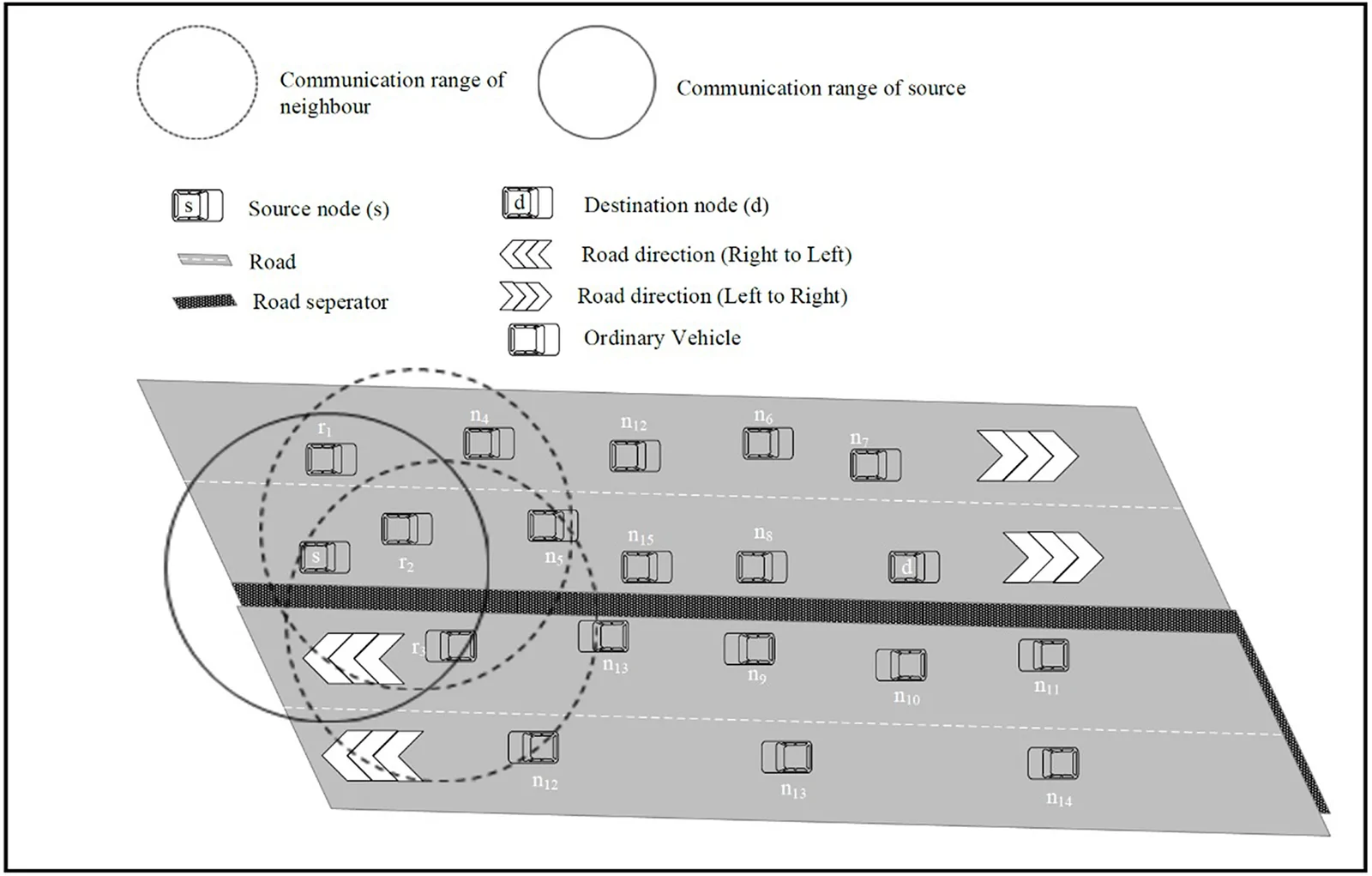
A bi-directional highway scenario for VANETs.

We propose Algorithm 1 that takes *s* and *d* as inputs, where *s* represents a source node and *d* refers to a destination node. Moreover, the output of Algorithm 1 includes success or failure during transmission of messages. Fig 2 depicts a highway scenario, where *s* intends to send a message *M* that is divided into a set of packets *P*, i.e., $P=\{p_{1},p_{2},p_{3},...,p_{z}\}$ with *z* representing the total number of packets. If *d* lies within the communication range of *s*, all the packets are forwarded straightaway. Contrarily, when *d* remains at multi-hop distance, *s* employs the services of intermediary relay nodes (herein, referred to as forwarder) for the transmission of *M* towards *d*. Suppose there are two possible routes to reach *d* from *s*, i.e., *X*_1_ and *X*_2_. *X*_1_ includes $s>>r_{3}>>n_{13}>>n_{9}>>n_{10}>>d$, whereas *X*_2_ remains $s>>r_{2}>>n_{5}>>n_{15}>>n_{8}>>d$. Here, *X*_1_ nodes bear opposite direction to *s* while nodes on *X*_2_ share the same direction. VANETs exhibit frequent topological changes due to high speed bi-directional movement of nodes, which makes selection of the best possible forwarder a challenging task. To this end, neighbours are identified using Algorithm 2, which is detailed in the following subsection.

### 3.2 Neighbour discovery

We propose Algorithm 2 that takes *N* as input, where *N* represents a set of all nodes. The proposed algorithm discovers neighbours and builds a neighbouring table (*R*). A neighbour refers to a node that lies within the communication range of *s*, hence, considered to be at a single-hop distance. When *s* intends to discover its neighbours, it broadcasts a *hello* message. All the nodes within its communication range respond with an acknowledgment (*ack*), where *ack* ensures the presence of a node at single-hop distance. Successful reception of *ack* creates an entry in $R_{s}$ for this node, which comprises Node Identity (*ID*), Current Position (*CP*), and Time Stamp (τ) of last successfully received *ack*. We assume that all nodes are equipped with Global Positioning System (GPS) for localization [31]. Here, τ is used to identify the most recently received *ack* and discards previous information. The output of Algorithm 2 remains *R*. Once the neighbours are identified, the process increments towards selections of the best forwarder among these neighbours. This selection remains the responsibility of our proposed Algorithm 3, which is detailed in the following subsection.

**Algorithm 2 Neighbour Discovery**

**Input:** *N*

**Output:** *R*

**Begin**

**Flush** $R_{s}$

*i* ⟵ 0

*s* broadcasts *hello* messages

*s* receives *ack*s from its neighbours

**Repeat**

*s* updates the $R_{s}$:

$R_{s}$ (*i*, 1) ⟵ *ID*

$R_{s}$ (*i*, 2) ⟵ *CP*

$R_{s}$ (*i*, 3) ⟵ τ

**Increment** *i*

**Until** successful processing of all the *ack*s

**Return** $R_{s}$

**End**

### 3.3 The best forwarder selection

A source may have multiple routes to reach a destination for the transmission of messages, however, nodes’ mobility produces frequent topological changes in ad hoc networks that makes the selection of route a challenging task. Such a decision becomes even more difficult on bi-directional highways in VANETs, where nodes exhibit high speed mobility [2,6,8]. A greedy algorithm [32] selects a forwarder that is nearest to its destination with respect to distance. However, such a distance-based selection remains incapble of catering for the frequent topological changes, where direction of a node plays a pivotal role. Hence, use of greedy protocols results in reduced link lifetime and network throughput, thereby, increasing packet losses and end-to-end delay. To this end, direction-based routing [2] takes into account the relative position of a source and a destination along with direction that significantly improves efficiency of a network. However, these protocols are not capable of managing traffic load on a selected path. This puts burden on the selected path that adversely impact the performance of a network, hence, rising the number of packet losses, increasing end-to-end delay, and reducing network throughput. To overcome this issue, our proposed protocol, i.e., PDBFS, introduces Algorithm 3 that takes *R* as input and finds the best forwarder.

**Algorithm 3 The Best Forwarder Selection**

**Input:** *R*

**Output:** *r_s_* and the best possible route to *d*

**Begin**

**For** i = 1 **To** *Size*(*R*)

$\eta (r_{i},d)$ ⟵ $\left|\overset{x}{\underset{i}{r}}-d^{x}\right|$ + $\left|\overset{y}{\underset{i}{r}}-d^{y}\right|$

$\kappa (r_{i},s)$ ⟵ $CaC(\theta_{r_{i}},\theta_{s})$

$\kappa (r_{i},s)⟵CAC\left\{Tan^{-1}\left(\frac{\overset{x1}{\underset{i}{r}}-\overset{x2}{\underset{i}{r}}}{\overset{y1}{\underset{i}{r}}-\overset{y2}{\underset{i}{r}}}\right),\;Tan^{-1}\left(\frac{s^{x1}-s^{x2}}{s^{y1}-s^{y2}}\right)\right\}$

**Try {**

**If** κ(s,d) remain the same **Then**

**If** *s* is rear and *d* is the front node **Then**

$\delta (r_{i},d)$ ⟵ $\frac{\eta (r_{i},d)}{\kappa (r_{i},s)}$, **If** $\kappa (r_{i},s)=0$ **Throw** “Exception Occurred” **End If**

**Else**

$\delta (r_{i},d)$ ⟵ $\eta (r_{i},d)$ $\kappa (r_{i},s)$

**End If**

**Else**

**If** *s* and *d* move away from each other **Then**

$\delta (r_{i},d)$ ⟵ $\eta (r_{i},d)$ $\kappa (r_{i},s)$

**Else**

$\delta (r_{i},d)$ ⟵ $\frac{\eta (r_{i},d)}{\kappa (r_{i},s)}$, **If** $\kappa (r_{i},s)=0$ **Throw** “Exception Occurred” **End If**

**End If**

**End If}**

**Catch (...){**$\delta (r_{i},d)⟵D_{Max}$**}**

$\rho_{r_{i}}=\rho_{max}-\left[\frac{\left\{\frac{\left(\frac{\upsilon_{d}-\upsilon_{r_{i}}}{\delta (r_{i},d)}\right)-\alpha }{\beta -\alpha }\right\}+\left(\frac{p_{c}}{P}\right)}{2}\right]$

**End For**

$\rho_{max}$ ⟵ $Max(\rho_{r})$

$\overset{s}{\underset{r}{\rho }}$ ⟵ $\rho_{max}$

**If** ($\overset{s}{\underset{r}{\rho }}$ ∈ $\omega_{3}$) **Then**

$p_{i}$>> $r_{s}$

*s* ⟵ $r_{s}$

**End If**

**If** ($\overset{s}{\underset{r}{\rho }}$ ∈ $\omega_{2}$) **Then**

$Mark\;(p_{i})$

$p_{i}$>> $r_{s}$

*s* ⟵ $r_{s}$

**End If**

**If** ($\overset{s}{\underset{r}{\rho }}$ ∈ $\omega_{1}$ **AND** $p_{i}$ is a safety message) **Then**

$p_{i}$>> $r_{s}$

*s* ⟵ $r_{s}$

**Else**

*s* finds an alternative route for the transmission of non-safety packet, i.e., $p_{i}$

*s* performs neighbour discovery

**End if**

**End**

In the given scenario depicted in Fig 2, Algorithm 2 produces $R_{s}$ comprising *r*_1_, *r*_2_, and *r*_3_ as the possible forwarders. We use Equation (1) to compute the Manhattan distance between each of the possible forwarders and *d* [33], as


$$\begin{array}{c} \eta (r_{i},d)⟵|\overset{x}{\underset{i}{r}}-d^{x}|+|\overset{y}{\underset{i}{r}}-d^{y}|, \end{array}$$ (1)

where η is the Manhattan distance between a possible forwarder $\left(r_{i}\right)$ and *d* with coordinates $\left(\overset{x}{\underset{i}{r}},\overset{y}{\underset{i}{r}}\right)$ and $\left(d^{x},d^{y}\right)$, respectively. PDBFS uses Manhattan distance instead of Euclidean distance, because nodes follow roads that are not always straight and include turns, thus, Manhattan distance gives a more realistic sense of separation and routing behavior in VANETs [5].

PDBFS does not consider η as the final distance, as it is not sufficient for message routing in a bi-directional scenario. For example, *r*_3_ lies at smaller distance than *r*_1_ and *r*_2_, therefore, a greedy protocol will select *r*_3_ as the best forwarder [34]. However, selection of *r*_3_ always remains a risk due to its opposite direction to the destination node *d*. Subsequently, there is a strong possibility of *r*_3_ going out of the range of the next forwarder, thereby, resulting in delivery failure of the message *M* to *d*. To address the aforementioned issue, we add a second parameter, i.e., direction component (θ). We propose a Classify and Compare function, *CaC*(.) function, which performs the classification and comparison. First, *CaC*(.) identifies the directions of nodes and then classify it with respect to the bi-directional highway using Equation (2), as


$$\begin{array}{c} \theta =\left\{ \begin{array}{cc} ⊃, & \forall \;0^{0}\leq \theta \leq 90^{0}\;OR\;270^{0}\leq \theta \leq 360^{0}\\ \subset , & \forall \;91^{0}\leq \theta \leq 270^{0} \end{array}\right., \end{array}$$ (2)

where ⊃ refers to the direction of nodes on the highway moving left to right and ⊂ represents the right to left direction of nodes with respect to the scenario shown in Fig 2. After evaluation of nodes directions, θ, *CaC*(.) proceeds towards its second task, i.e., evaluation of relative direction (κ) among nodes. To this end, we propose Equation (3) that computes κ, as


$$\begin{array}{c} \kappa (r_{i},s)⟵CAC(\theta_{r_{i}},\theta_{s}). \end{array}$$ (3)

Equation (3) is further be extended as


$$\begin{array}{c} \kappa (r_{i},s)\leftarrow CAC\left\{(Tan^{-1}\left(\frac{\overset{x1}{\underset{i}{r}}-\overset{x2}{\underset{i}{r}}}{\overset{y1}{\underset{i}{r}}-\overset{y2}{\underset{i}{r}}}\right),\;Tan^{-1}\left(\frac{s^{x1}-s^{x2}}{s^{y1}-s^{y2}}\right)\right\}, \end{array}$$ (4)

which evaluates κ between a possible forwarder $r_{i}$ and a source node *s*. Here, coordinates for $r_{i}$ and *s* remain (*x*1, *y*1) and (*x*2, *y*2) at two consecutive time steps (i.e., $\tau_{0}$ and $\tau_{1}$), respectively. The *CaC*(.) function returns a binary value that is assigned to κ. Here, a zero (0) indicates that nodes bear different direction, whereas the value one (1) means that nodes share the same direction. Furthermore, PDBFS takes into the account relative positions of source and destination nodes to evaluate the final distance (δ) between a possible forwarder and a destination node [9]. This leads towards four different cases, which are as under.

1. When *s* remains rare to *d* and both *s* and *d* share the same direction, δ is computed using Equation (5) [9], as
  $$\begin{array}{c} \delta (r_{i},d)⟵\frac{\eta (r_{i},d)}{\kappa (r_{i},s)}. \end{array}$$ (5)
2. When *s* remains the front node, *d* is the rare node, and both share the same direction, δ is computed using Equation (6) [9], as
  $$\begin{array}{c} \delta (r_{i},d)⟵\eta (r_{i},d)\kappa (r_{i},s). \end{array}$$ (6)
3. When *s* and *d* bear opposite directions and they are towards each other, δ is computed using Equation (5).
4. When *s* and *d* bear opposite directions and both of these nodes are moving away from each other, δ is computed using Equation (6).

It is worth-mentioning that during computation of δ, division by zero (0) produces an exception (i.e., runtime error), which is the main feature and novelty of our proposed Algorithm 3. When such an exception occurs, the algorithm takes a predefined value, i.e., $D_{Max}$, as the distance between the nodes that may vary in different scenarios. For example, $D_{Max}$ may be taken as the distance between farthest cities in a certain country or the farthest ends of a highway. Since the probability of link and packet losses increase with the selection of a forwarder moving in opposite direction, Algorithm 3 reduces packet losses and provides better communication by avoiding such selection.

Selection of a route for the transmission of messages on such a direction-aware criterion yields significantly improved performance by minimizing the packet loss ratio and end-to-end delay and increases the network throughput. However, this may put burden on a selected path, as all the messages are supposed to follow it. This may result in congestion that adversely impact the network performance. Relative speed among nodes retains a pivotal role to enhance link lifetime, which has not been used in message routing by any protocol in the literature to the best of our knowledge. To this end, we pioneer the use of relative speed of nodes for the selection of the best possible forwarder, as speed of a node has a major impact on link reliability. For example, we consider two nodes, *r*_2_ and *n*_4_, that are moving in the same direction bearing the speeds 18 meters per second (m/s) and 34 m/s, respectively. Since relative speed between the nodes remains inversely proportional to the link lifetime, *n*_4_ will go out of the range of *r*_2_ very quickly, thereby, resulting a link breakage. Therefore, lifetime of a link strongly remains dependent upon the relative speed of the corresponding nodes.

Since increased link lifetime ensures reliable delivery of messages, we introduce a novel technique for probability computation of a possible forwarder. PDBFS is a pioneering protocol to include two new parameters in this regard, namely, relative speed among nodes and packet rate, for the selection of the best forwarder. Relative speed enables PBDFS to efficiently manage the topological changes, whereas packet rate prevents the route from getting overloaded. This yields minimized packet loss rate, reduced end-to-end delay, and increased link lifetime and network throughput for PDBFS (as shall be discussed in Section 4). To find the best forwarder, probability of each possible forwarder ($\rho_{r_{i}}$) is computed. To this end, we derive a new probability computation equation, as


$$\begin{array}{c} \rho_{r_{i}}=\rho_{max}-\left[\frac{\left\{\frac{\left((\upsilon_{d}-\upsilon_{r_{i}})+(\frac{\delta (r_{i},d)}{\tau })\right)-\alpha }{\beta -\alpha }\right\}+\left(\frac{p_{c}}{P}\right)}{2}\right], \end{array}$$ (7)

where $\rho_{max}$ represents the maximum probability for successful packet delivery. $\upsilon_{r_{i}}$ and $\upsilon_{d}$ refer to the corresponding speeds of a possible forwarder and the destination, respectively. τ is the time step. α and β denote the minimum and and maximum values of the fractional part $\left((\upsilon_{d}-\upsilon_{r_{i}})+(\frac{\delta (r_{i},d)}{\tau })\right)$ in the Equation (7), respectively. Furthermore, $p_{c}$ counts the number of packets forwarded to a certain possible forwarder, thus, $\left(\frac{p_{c}}{P}\right)$ in Equation (7) evaluates the aforementioned packet rate.

After computation of $\rho_{r_{i}}$ for each element of the set *R*, Algorithm 3 of our proposed PDBFS protocol uses a *Max*(.) function to find the highest probability node ($\rho_{max}$) in the set ($\rho_{r}$), as


$$\begin{array}{c} \rho_{max}⟵Max(\rho_{r}), \end{array}$$ (8)

where $\rho_{r}$ includes individual probabilities of all the possible forwarders in set *R*. A node bearing the highest probability is selected as the next forwarder, i.e., $r_{s}$ with probability of $\overset{s}{\underset{r}{\rho }}$, as


$$\begin{array}{c} \overset{s}{\underset{r}{\rho }}⟵\rho_{max}. \end{array}$$ (9)

The aforementioned computed probability $\overset{s}{\underset{r}{\rho }}$ is used to check the appropriateness of the selected forwarder $r_{s}$ for the delivery of a message *M*. Messages are of two types in VANETs, namely safety and non-safety messages. A safety message is used to intimate a node regarding a possible accident with another node, traffic congestion or blockage on road, etc. Contrarily, non-safety messages are used for entertainment or infotainment purposes, e.g., a person listening to a song can share it with another node using non-safety messages [2]. Therefore, safety messages require reliable transmission and are time-sensitive. For example, a safety message intimating a node, regarding a possible collision with another node, needs to ensure message delivery success with reduced end-to-end delay for the prevention of nodes against the collision. This makes metrics, such as messages drop ratio, end-to-end delay, network throughput, and link lifetime more critical for safety messages than non-safety messages. Hence, safety messages are required to be treated on higher priority in comparison with non-safety messages. Both greedy and direction-based protocol lack this functionality that remains extremely important in VANETs. In this regard, our proposed PDBFS introduces a new technique to prioritize safety messages. We employ uniform probability distribution [35] that divides the probability of a forwarder into three different levels of ω, i.e., $\omega_{1}$, $\omega_{2}$, and $\omega_{3}$. Here, $\omega_{3}$ refers to the most reliable range that ensures in-time delivery of packets, whereas $\omega_{1}$ denotes the most inferior range in this regard. If $\overset{s}{\underset{r}{\rho }}$ falls in the range of $\omega_{3}$, a packet is forwarded straightaway. Similarly, for the value of $\overset{s}{\underset{r}{\rho }}$ that ranges within $\omega_{2}$, the packet is marked to the source and then forwarded to $r_{s}$. This intimates a source regarding risks in transmission of packets on the selected path. Finally, when $\overset{s}{\underset{r}{\rho }}$ lies within the most risky range, i.e., $\omega_{1}$, for packets delivery, only safety messages are transmitted and all the non-safety messages are dropped. For the definition of the aforementioned probability levels, we propose


$$\begin{array}{c} \omega =\left\{ \begin{array}{cc} \omega_{1}, & \forall \;0.00\leq \omega_{1}\leq 0.33\\ \omega_{2}, & \forall \;0.34\leq \omega_{2}\leq 0.66\\ \omega_{3}, & \forall \;0.67\leq \omega_{1}\leq 1.00 \end{array}\right., \end{array}$$ (10)

Algorithm 1 through Algorithm 3 constitutes our proposed PDBFS protocol. This protocol enables reliable and in-time delivery of safety messages with minimized packet loss ratio, reduced end-to-end delay, increased link lifetime and enhanced network throughput. Furthermore, PDBFS introduces a novel best forwarder probability estimation technique. Moreover, our proposed protocol pioneers the use of relative speed among nodes and packet rate as parameters to compute probability of the best forwarder. This work also proposes a new technique to prioritize safety over non-safety messages. The following section demonstrates the efficacy of our proposed protocol.

## 4 Performance evaluation

This section evaluates the performance of our proposed PDBFS protocol in comparison with an eminent protocols from the literature, namely, reliable Target Driven and Mobility Prediction-based routing (TDMP) [12], Ad hoc On-Demand Distance Vector-Reliable (AODV-R) [13] and Traffic Density Stable Routing Protocol based on Connection and Distance (TDSRP-DC) [14]. A brief discussion of the parameters employed in the simulations is given below.

### 4.1 Simulation setup

Simulation results are derived using Network Simulator (ns-2, version 2.35), which is widely used by state-of-the-art [5,12,36–39]. Unless otherwise stated, all simulations are based on the scenario depicted in Fig 2. Table 1 lists the parameters used, where simulations area is taken as 2500 *m*^2^ employing the TwoRayGround propagation model. Two-Ray Ground is well-suited for highway VANETs because it explicitly models the dominant line-of-sight plus ground-reflection propagation that characterizes freeways or highway environments. Unlike stochastic geometric models, such as Nakagami fading model, it provides deterministic and stable signal estimation without requiring parameter tuning. It is also computationally simpler, making it efficient for large-scale simulations with large number of vehicles. In unobstructed highways, it avoids the over-randomization introduced by fading models, yielding more consistent and interpretable results [40]. Moreover, nodes are taken in the range of 0–600 that are deployed randomly and each nodes is equipped with omni-directional antenna. These nodes are classified into sparse, medium, and dense networks with respect to nodes’ density [9]. Furthermore, movement of nodes remains bi-directional bearing speeds ranging from 0 *m*/*s* to 42 *m*/*s*. Results presented are averaged over 25 replicated simulation runs by keeping all parameters fixed and changing the random seed values. Simulation are conducted with respect to the performance evaluation metrics detailed in the following subsection.

**Table 1:** Simulation parameters.

Parameter	Configuration
Area of Simulation	2500 m^2^
Number of Nodes	0–600
Speed of nodes	0 *m*/*s* – 42 *m*/*s*
Kind of Traffic	Bi-directional
Propagation Model	TwoRayGround
Number of Lanes	6
Lane Width	3.5−3.75 m
Vehicle headway	1.5–2s (Time Gap) 20–40m (Distance Gap) at moderate speeds
Transport Layer	TCP
MAC Layer	IEEE 802.11
Simulation Time	$3*10^{5}$ ms

### 4.2 Performance evaluation metrics

Packet loss rate $\left(P_{l}\right)$, end-to-end-delay $\left(E_{d}\right)$, network throughput $\left(T_{p}\right)$, and link lifetime are taken as metrics to evaluate the performance. These metrics are used by state-of-the-art to investigate the performance of VANETs routing protocol for the delivery of messages [5,30].

1. **Packet Loss Rate (**$P_{l}$**):** This metric is defined as the ratio of dropped packets to the total number of packets transmitted. $P_{l}$ is computed using Equation (11) [9] as,
  $$\begin{array}{c} P_{l}=\frac{\sum_{i=1}^{P_{t}}P_{d_{i}}}{P_{t}}, \end{array}$$ (11)
  where $P_{l}$ refers to packet loss rate, $P_{d_{i}}$ represents a dropped packet, and $P_{t}$ denotes total number of packets transmitted across the network.
2. **End-to-End delay (**$E_{d}$**):** Average end-to-end delay refers to the ratio between delays experienced during successful transmission of an individual packets ($E_{s_{i}}$) and the total number of packets received at destination nodes ($P_{s}$). $E_{d}$ is computed using Equation (12) [9], as
  $$\begin{array}{c} E_{d}=\frac{\sum_{i=1}^{E_{p}}E_{s_{i}}}{P_{s}}. \end{array}$$ (12)
3. **Network Throughput (**$T_{p}$**):** This metric refers to the ratio of successful delivery of received packets to the total number of packets transmitted across the network. $T_{r}$ is computed using Equation (13) [9], as
  $$\begin{array}{c} T_{p}=\frac{\sum_{i=1}^{P_{t}}P_{s_{i}}}{P_{t}}. \end{array}$$ (13)
4. **Link Lifetime:** This metric refers to the time, in terms of milliseconds (ms), where two nodes remain within the communication range of each other. Thus, the time elapsed indicates the active status of a link.

### 4.3 Results analysis

This section presents and discusses the simulation results obtained using the aforementioned setup and performance evaluation metrics. The generated results are thoroughly analyzed to evaluate the effectiveness of the proposed protocol under varying network densities. Furthermore, a comparative performance assessment with state-of-the-art protocols is provided to demonstrate the efficacy of the proposed approach in terms of reduced packet losses, minimized end-to-end delay, improved network throughput, and enhaced link lifetime.

#### 4.3.1 Packet loss rate.

Packet loss rate remains an important metric to evaluate the performance of routing protocols. Since packet loss rate stands inversely proportional to the network efficiency, high packet loss rate adversely affects the performance of a certain protocols. Hence, an efficient routing protocol is bound to reduce packet losses on the network to the maximum possible extent [2,5,6,8,9]. Network density has a major impact upon such losses, where network density remains proportional to the packet loss rate. Thus, we evaluate the performance of our proposed PDBFS protocol along with TDMP, AODV-R, and TDSRP-DC on variant network densities. To this end, we employ different network categories with respect to nodes’ density, i.e., sparse, medium, and dense.

**Fig 3:**
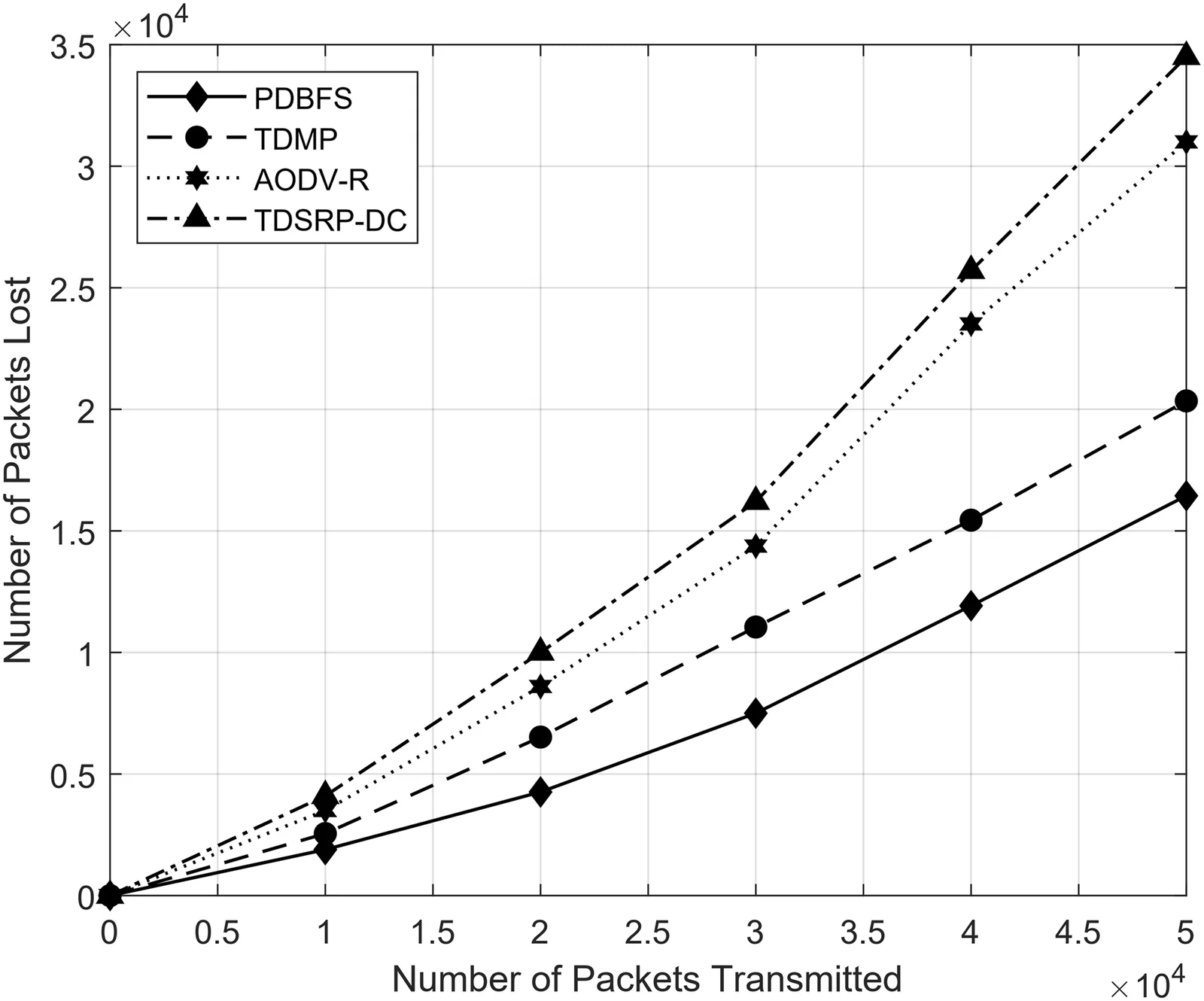
Average packet loss rate during transmission of safety messages for sparse network.

**Fig 4:**
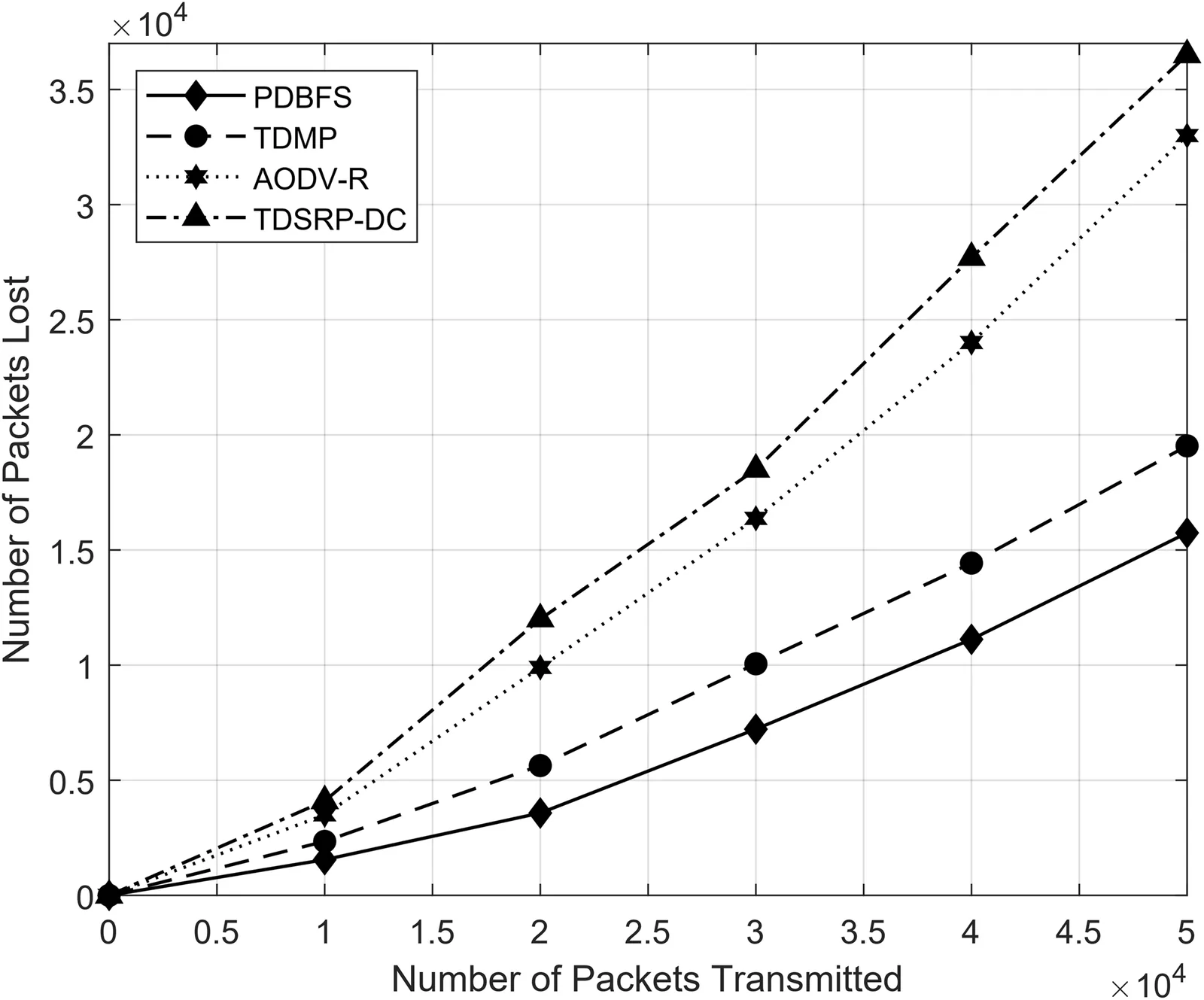
Average packet loss rate during transmission of safety messages for medium network.

**Fig 5:**
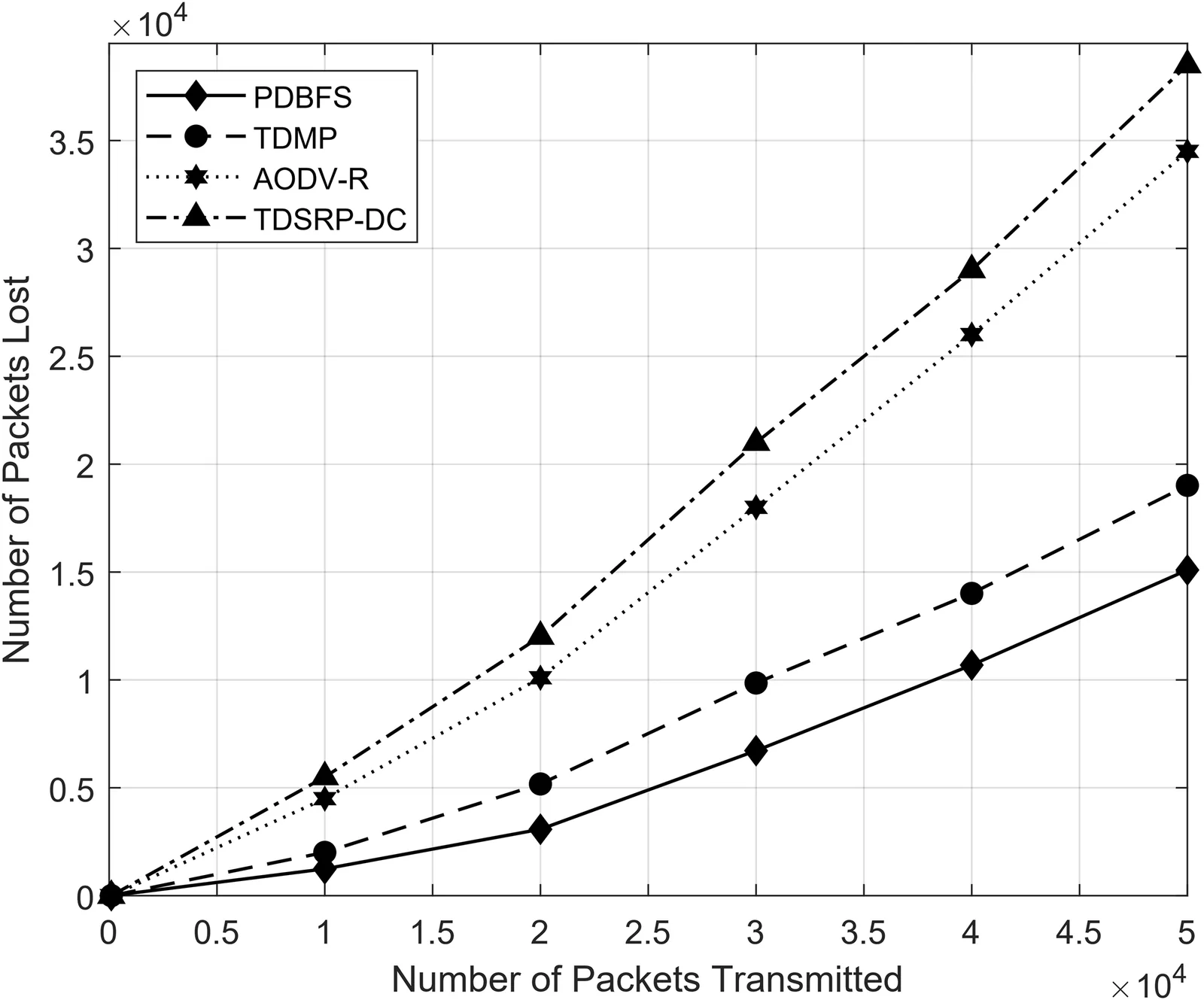
Average packet loss rate during transmission of safety messages for dense network.

Simulation results, generated in terms of packet loss rate, for the aforementioned protocols are presented in Figs 3–5 that maps onto the sparse, medium, and dense networks, respectively. Increased source nodes on the network rises congestion on the network. A congested network exhibits higher packet loss rate, as shown in Figs 3–5. Moreover, it can be seen that PDBFS exhibits considerably reduced packet loss rate in comparison with TDMP, AODV-R, and TDSRP-DC, thus, yielding improved performance for all the categories of networks. This is because, TDMP, AODV-R, and TDSRP-DC lack the ability to cater for the frequent topological changes and communication overhead. To this end, our proposed PDBFS proposes the use of two additional parameters, namely, relative speed of a node and communication load, to evaluate the probability for the selection of the best forwarder. Relative speed of a node plays a crucial role in link lifetime and in ensuring in-time delivery of messages. This work stands as a pioneering approach that takes relative speeds of nodes into account for the selection of the best forwarder that selects a route with highest link lifetime. Considering the scenario depicted in Fig 2, suppose *n*_4_ is the front node and traveling with a speed twice as the speed of its rear node, *r*_2_. Since relative speed among nodes remains inversely proportional to the link lifetime, *n*_4_ is expected to leave the communication range of *r*_2_ soon, thereby, resulting a link breakage. To avoid this, PDBFS considers relative speed of nodes that helps to select a link with higher lifetime. Moreover, PDBFS uses packet rate that prevents a route from getting overloaded. A node keeps track of the packets forwarded through it, as detailed in Section 3.3. Probability of each possible forwarder is computed and a node having the highest probability is selected as the best forwarder. This enables PDBFS to achieve reduced packet losses in comparison with TDMP, AODV-R, and TDSRP-DC, thereby, ensuring reliable transmission of safety messages.

#### 4.3.2 End-to-end delay.

Safety message are time-sensitive application, whereas delay does not have a significant impact upon non-safety messages [2,5,6,8,9]. Therefore, end-to-end delay retains a vital role in determining the performance of routing protocols.

**Fig 6:**
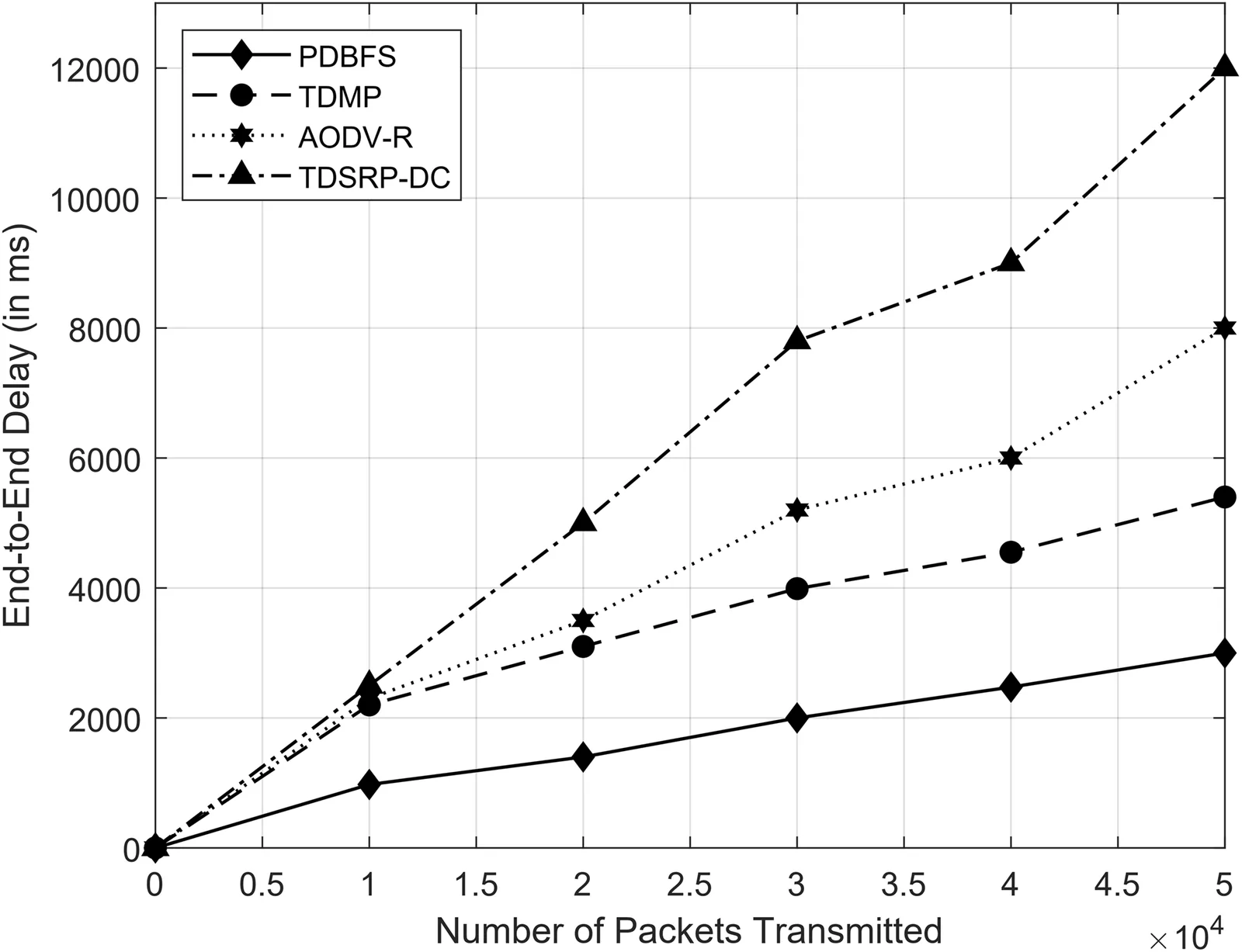
End-to-end delay experienced during transmission of safety messages for sparse Network.

**Fig 7:**
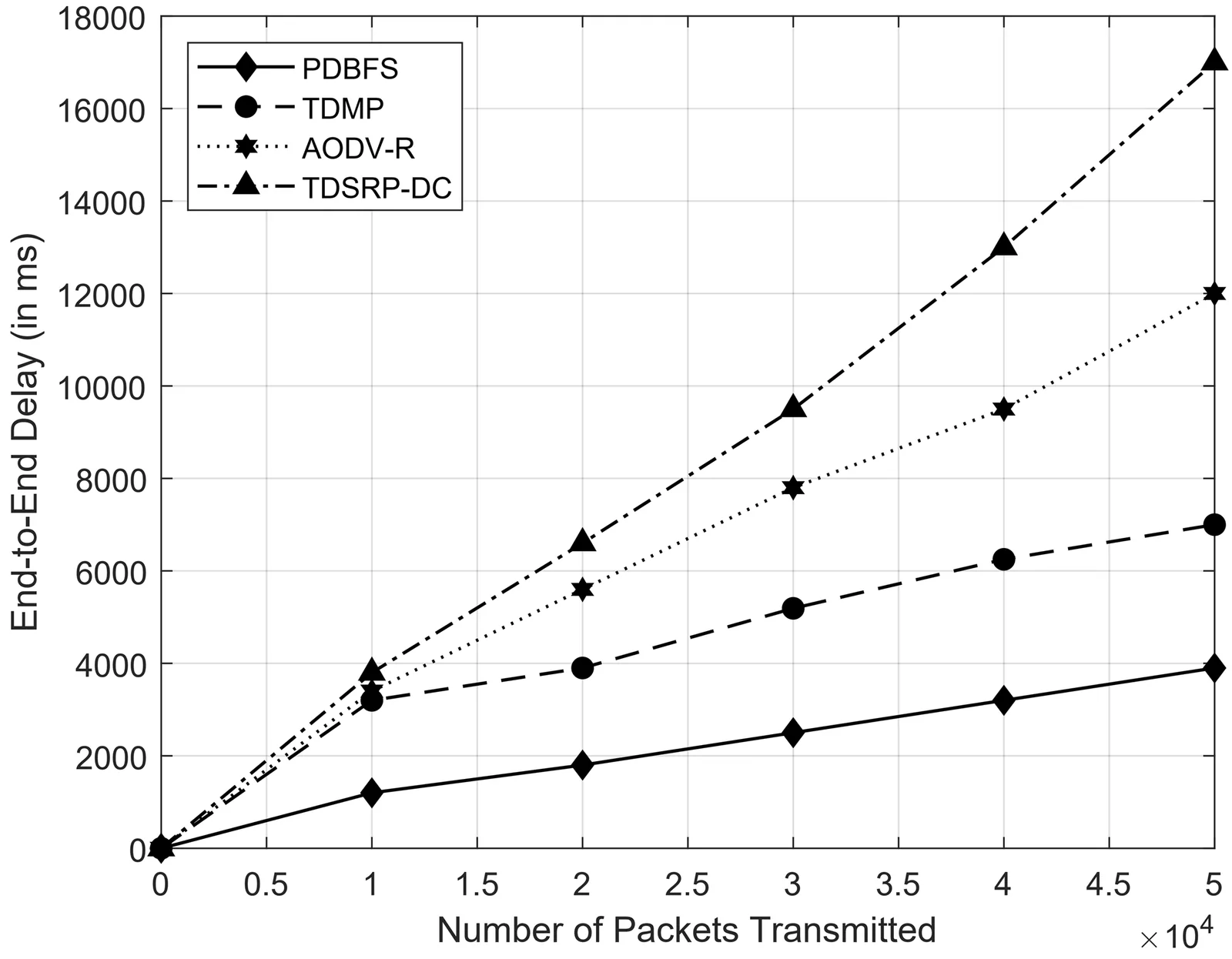
End-to-end delay experienced during transmission of safety messages for medium Network.

**Fig 8:**
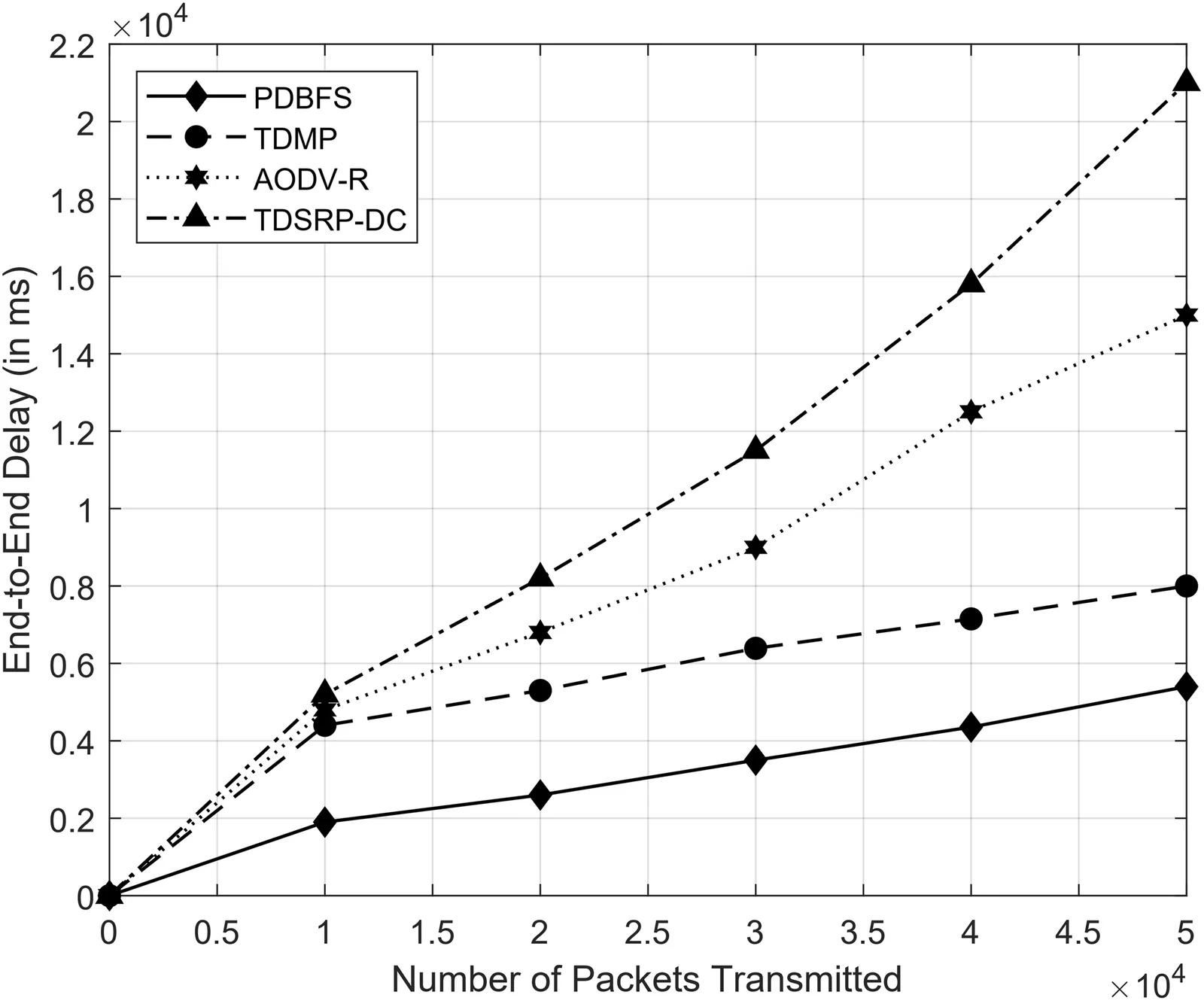
End-to-end delay experienced during transmission of safety messages for dense Network.

Simulation results, generated in terms of end-to-end delay, for the aforementioned protocols are presented in Figs 6–8 that maps onto the sparse, medium, and dense networks, respectively. In all the three categories of networks, it can be seen that PDBFS shows significantly reduced end-to-end delay as compared to TDMP, AODV-R, and TDSRP-DC, thereby, providing improved efficiency. PDBFS introduces a new parameter, i.e., packet rate. A node computes its packet rate, which is used to calculate the probability for its selection as the best forwarder. Here, packet rate remains inversely proportional to the probability of a node to become the next forwarder, as discussed in Section 3.3. Selection of a route with smaller congestion ensures better performance for PDBFS in comparison with TDMP, AODV-R, and TDSRP-DC that do not provide any means for traffic load management on a selected route.

#### 4.3.3 Network throughput.

**Fig 9:**
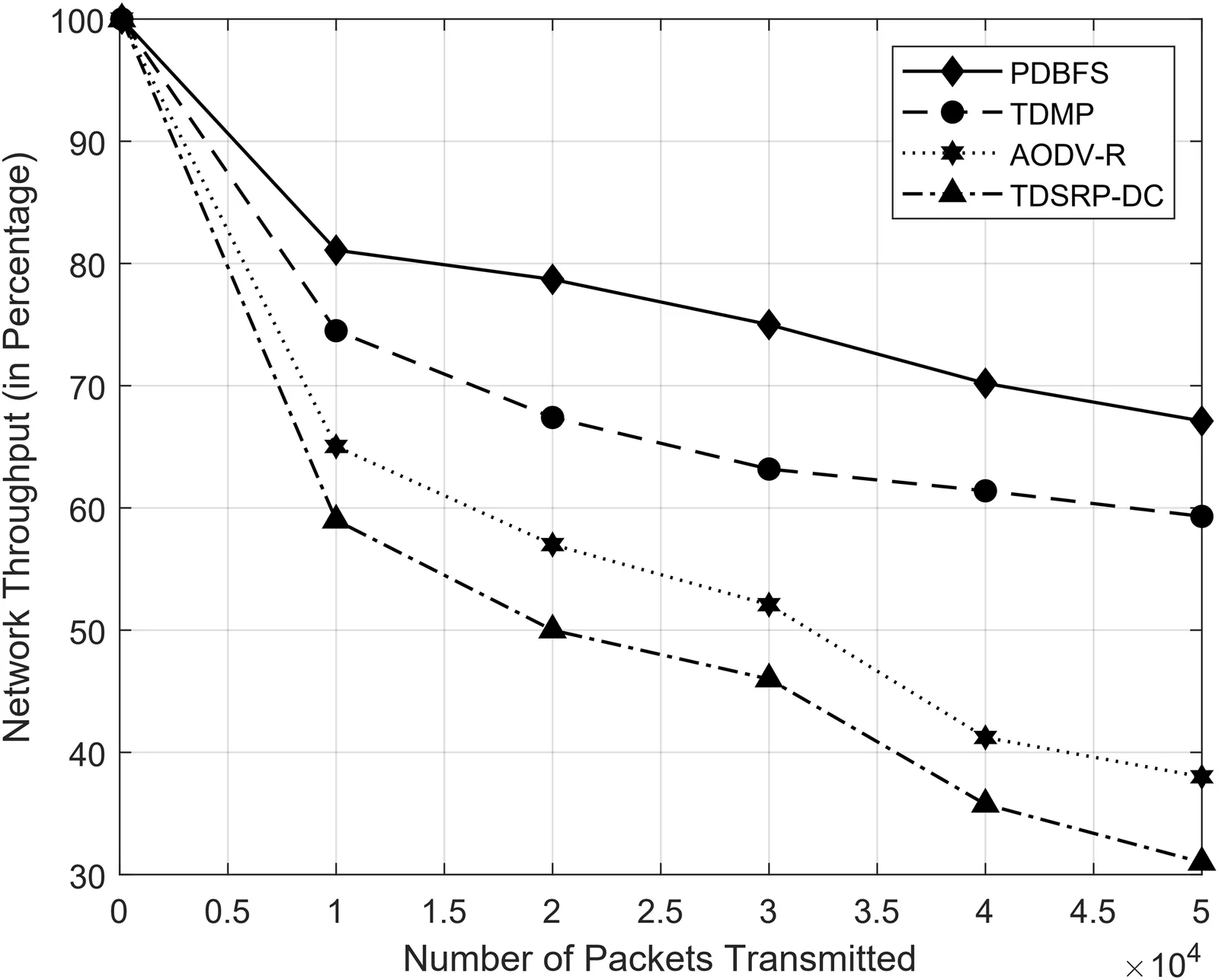
Network throughput achieved during transmission of safety messages for sparse Network.

**Fig 10:**
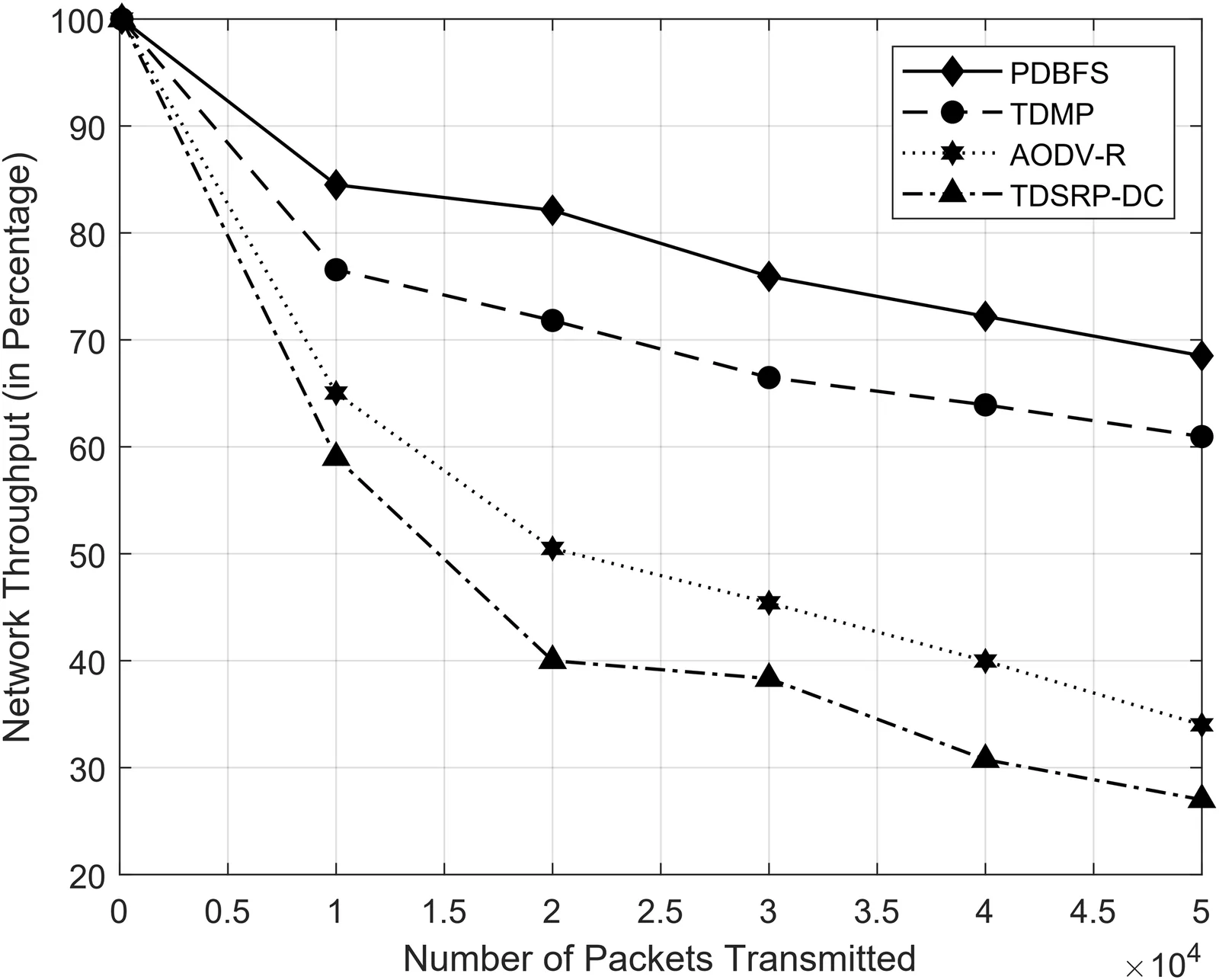
Network throughput achieved during transmission of safety messages for medium Network.

**Fig 11:**
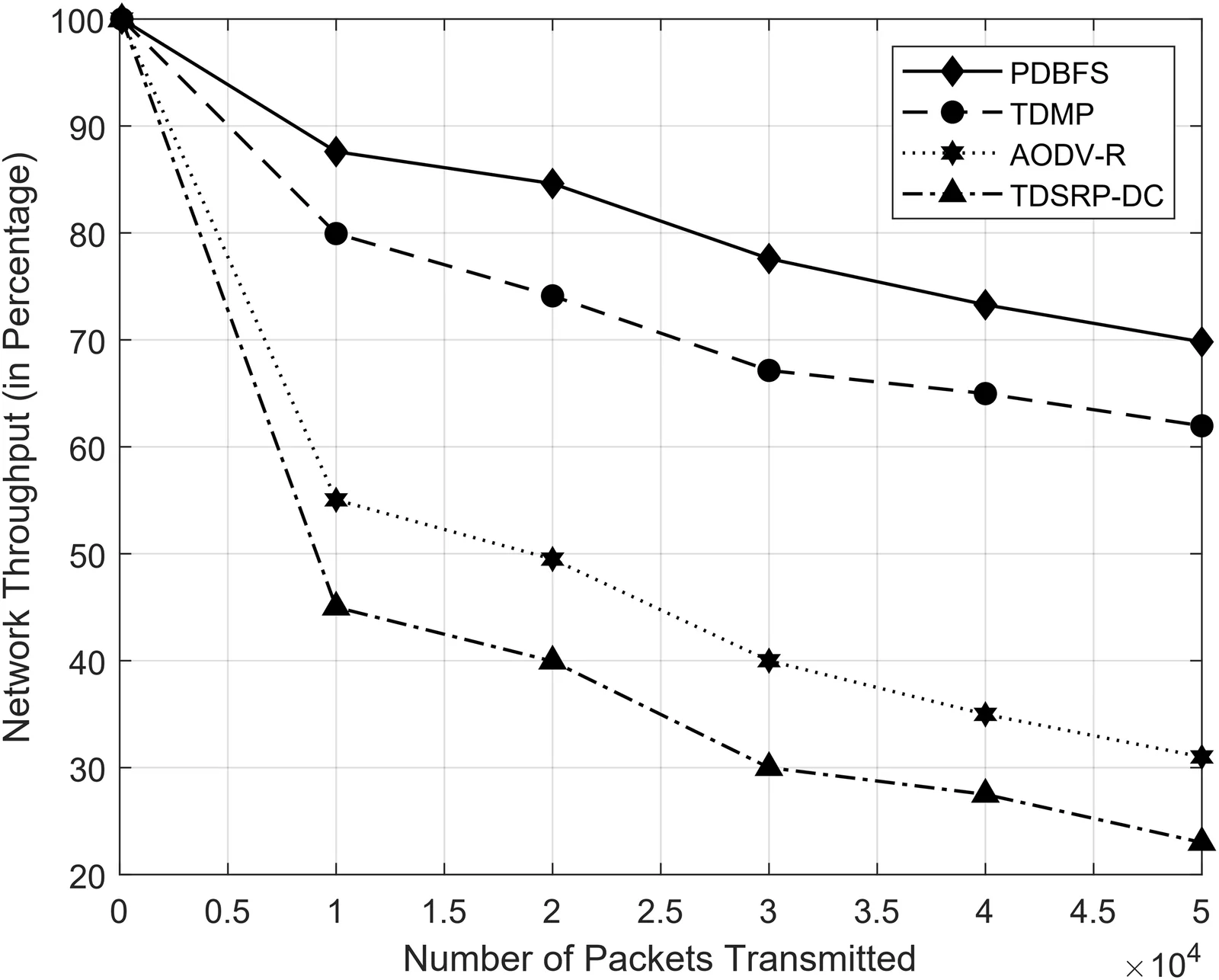
Network throughput achieved during transmission of safety messages for dense Network.

Network throughput is also among the important metrics to evaluate the performance of routing protocols [2,6,9]. Simulation results, generated in terms of network throughput, for the aforementioned protocols are presented in Figs 9–11 that maps onto the sparse, medium, and dense networks, respectively. PDBFS outperforms TDMP, AODV-R, and TDSRP-DC with a significant margin by increasing the network throughput, hence, showing enhanced efficiency for all the aforementioned network categories. This achievement of PDBFS resulted due to the addition of two new parameters (i.e., relative speed and packet rate), in addition to direction-aware greedy parameters, for the selection of the best forwarder. Relative speed tackles the topological changes, whereas packet rate prevents the link from getting congested. Hence, producing increased network throughput.

#### 4.3.4 Link lifetime.

**Fig 12:**
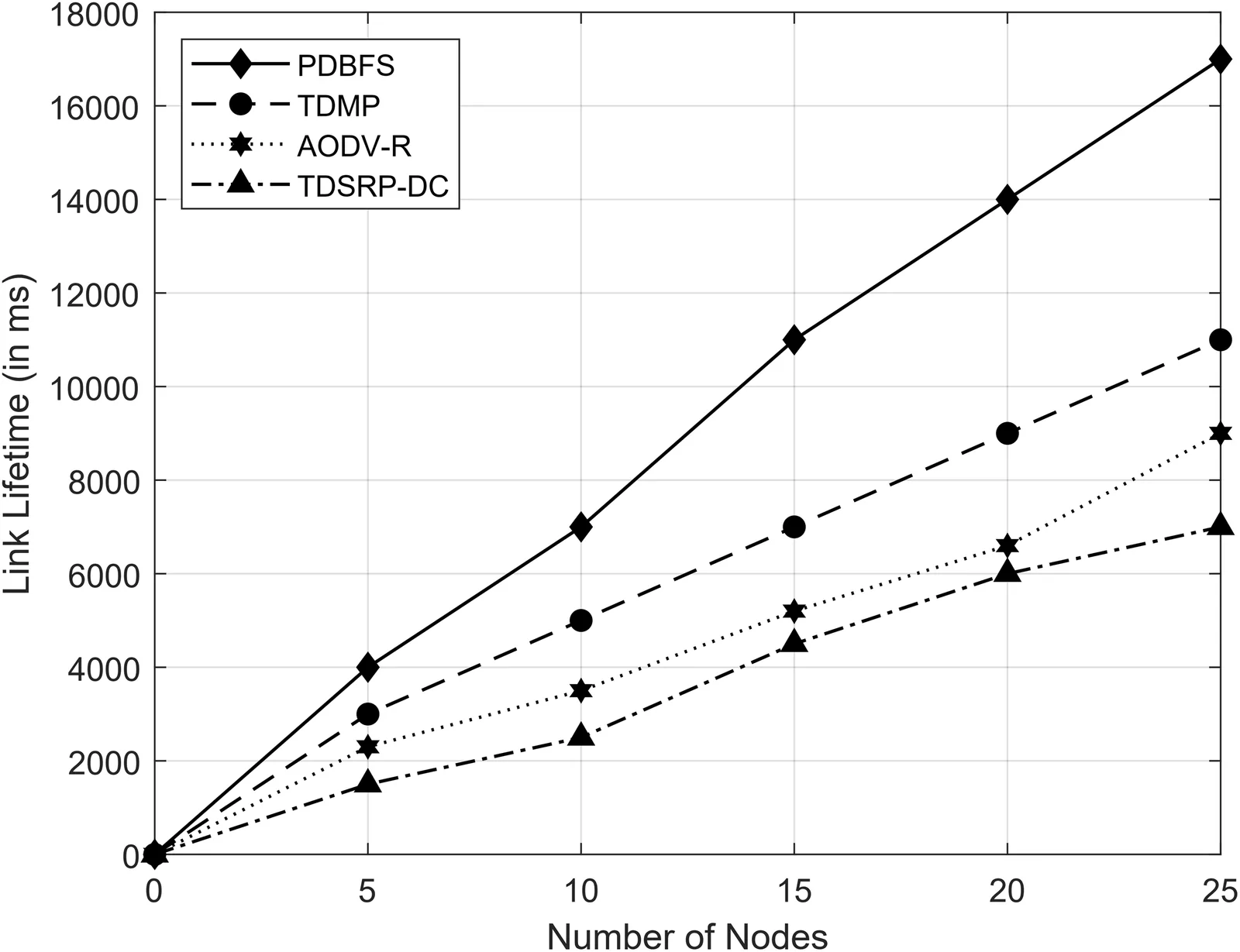
Link lifetime among nodes.

Link lifetime is one of the major parameters to ensure reliable delivery of messages. Selection of a route with shorter lifetime may cause packet drops at a higher rate. Link lifetime increases with the increase of nodes’ density, as shown in Fig 12. Here, relative speed among nodes remains critical that is inversely proportional to the link lifetime, which can be exploited to avoid link breakages. Due to neglection of this critical metric, TDMP, AODV-R, and TDSRP-DC remain incapable of catering for the frequent topological changes in VANETs. Contrarily, PDBFS employs the aforementioned parameter for the selection of the best forwarder. This results in selection of route with higher lifetime that reduces packet losses, thereby, ensuring reliable transmission of safety messages. Simulation results, presented in Fig 12, conforms our aforementioned claim, where PDBFS achieves significantly increased link lifetime in comparison with eminent protocols, such as TDMP, AODV-R, and TDSRP-DC.

### 4.4 Critical discussion

This work proposes a novel protocol, namely PDBFS, which is a probabilistic approach to enhance routing of safety messages between nodes on highways. This section presents a summary of the simulation results generated for the performance evaluation of PDBFS in comparison with eminent protocols from the existing body of knowledge. Furthermore, this section includes limitation and significance of our proposed protocol.

#### 4.4.1 Simulation results summary.

Packet loss rate remains inversely proportional to reliable transmission of safety messages. Simulation results demonstrated in Section 4.3.1 prove that PDBFS reduces the packet loss rate by 6.2%, 18.1%, and 23.4% in comparison with TDMP, AODV-R, and TDSRP-DC, respectively, that affirms our claim of improved reliability. Link lifetime is another factor in this regard that remains proportional to reliability. Simulation results, presented in Section 4.3.4, show that our proposed PDBFS protocol improves the link lifetime by 3600 ms, 4101 ms, and 4307 ms in comparison with TDMP, AODV-R, and TDSRP-DC, respectively, which further validate its efficacy. Moreover, end-to-end delay affects the in-time delivery of safety messages, where increased delay adversely impact the performance of an accident prevention application. PDBFS minimizes this delay by 2388 ms, 2914 ms, and 3362 ms in comparison with TDMP, AODV-R, and TDSRP-DC, respectively, as shown in Section 4.3.2. Similarly, enhanced network throughput ensures successful delivery of safety messages. Results depicted in Section 4.3.3 conforms to our claim, where PDBFS increases network throughput by 9.3%, 15.3%, and 22.0% in comparison with TDMP, AODV-R, and TDSRP-DC, respectively.

#### 4.4.2 Statistical analysis of the simulation results.

Table 2 shows statistical analysis of the simulation results, which are briefly discussed below.

1. **Mean:** Mean refers to the average value of the collected simulation data and represents the overall trend of the results [41]. In Table 2, it can be observed that when the network changes from sparse to dense, the average throughput increases while packet loss decreases. This indicates that a denser network improves communication efficiency and overall performance.
2. **Confidence Interval:** A Confidence Interval shows the range within which the true value of the result is likely to fall with a certain level of confidence [41]. The intervals in Table 2 are very narrow, which suggests that the simulation results are consistent and dependable. This means that if the simulation is repeated, the results would likely remain close to the reported values.
3. **Variance:** Variance measures how much the values in the data differ from the average value [41]. In Table 2, the dense network has a lower variance compared to sparse and medium networks, indicating that the results are more stable and less scattered. This suggests that performance becomes more consistent as the network density increases.
4. **Convergence:** Convergence indicates how quickly the simulation results settle to stable values during the simulation process [41]. The lower convergence values, shown in Table 2, for the dense network show that the simulation stabilizes faster compared to sparse and medium networks. This reflects better reliability and faster stabilization of results in dense network conditions.
5. **Monte Carlo Error:** Monte Carlo error represents the level of uncertainty that arises due to the random nature of simulation-based experiments [41]. The relatively small error values in Table 2 indicate that the simulation results are accurate and trustworthy.

**Table 2:** Statistical analysis of the simulation results.

Network Type	Metrics	Mean	Confidence Interval	Variance	Convergence	Monte Carlo Error
Sparse	Throughput	65.2	64.89 - 65.92	2.48	0.024	0.25
	Packet Loss Rate	34.8	34.32 - 35.42	2.48	0.045	0.25
Medium	Throughput	68.02	67.92 - 68.87	1.79	0.020	0.21
	Packet Loss Rate	32.17	31.72 - 32.96	1.79	0.041	0.21
Dense	Throughput	70.2	69.35 - 70.40	1.21	0.016	0.16
	Packet Loss Rate	30.25	29.53 - 30.57	1.21	0.036	0.16

#### 4.4.3 Limitation.

PDBFS may suffer performance degradation due to various inherent limitations of GPS, including privacy preservation constraints, intermittent signal failures (especially in indoor or dense urban environments), and localization inaccuracies caused by multipath effects or atmospheric disturbance. These issues may lead to unreliable positioning information, ultimately affecting the efficiency and robustness of a system. To address these challenges, we plan to enhance the proposed PDBFS with GPS-less localization techniques, which may provide more stable and accurate location estimation even in GPS-denied areas, thereby, improving overall performance and reliability.

#### 4.4.4 Research significance.

Recent technological developments are transforming the legacy highways into ITS. An ITS converts an ordinary node into intelligent one, which are capable of automated environmental learning. PDBFS is an effort to ensure reliable in-time delivery of the aforementioned learnt information through safety messages. This will minimize road accidents, hence, ensuring safer journeys.

## 5 Conclusion and future work

Road accident is one of the major causes of deaths around the world. In this regard, ITS injects intelligence into the nodes, which is exploited to prevent road accidents. To this end, VANETs equip ITS with several applications that predict and intimate nodes to apply the preventive measures. Here, accuracy in prediction is not the only parameter to ensure safety from accidents, rather in-time reliable also stands critical. Considering the time-sensitive nature of these applications, the existing literature provides numerous protocols. However, these protocols are incapable of managing frequent topological changes in VANETs. Moreover, lack of effective traffic load management also degrade their performance. To address these issues, we propose a novel protocol, namely PDBFS. This work proposes a new probabilistic approach for the selection of the best forwarder. PDBFS pioneers the use of relative speed of nodes and packet rate in addition to existing parameters of direction-aware greedy protocols. Relative speed helps to manage frequent topological changes, whereas packet rate accomplishes efficient load management. Furthermore, this work introduces a new method to prioritize the time-critical safety messages over non-safety messages. Simulation results demonstrate that PDBFS reduces end-to-end delay, minimizes packet loss rate, enhances network throughput and improves link lifetime in comparison with eminent VANET routing protocols. PDBFS enables safety messages routing on highways, which can be extended for urban scenarios that comprises intersections. Also, GPS-less localization can be considered as future extension of this work. Additionally, further insightful statistical analysis leads towards pattern and uncertainty quantification of data, which can be used for the optimization of routes.
